# 4D flow cardiac magnetic resonance imaging reveals ventricular dyssynchrony and intracardiac flow alterations in an infant with Wolff–Parkinson–White syndrome

**DOI:** 10.1093/ehjcr/ytag469

**Published:** 2026-06-17

**Authors:** Hideharu Oka, Yuki Shibagaki, Kouichi Nakau

**Affiliations:** Department of Pediatrics, Asahikawa Medical University, 2-1-1-1, Midorigaoka-Higashi, Asahikawa, Hokkaido 078-8510, Japan; Department of Pediatrics, Asahikawa Medical University, 2-1-1-1, Midorigaoka-Higashi, Asahikawa, Hokkaido 078-8510, Japan; Department of Pediatrics, Asahikawa Medical University, 2-1-1-1, Midorigaoka-Higashi, Asahikawa, Hokkaido 078-8510, Japan

**Keywords:** Dyssynchrony, 4D flow CMR, Intracardiac flow, Wolff–Parkinson–White syndrome

## Summary

We report a case of Wolff–Parkinson–White syndrome evaluated using 4D flow cardiac magnetic resonance imaging (CMR). The findings suggest that 4D flow CMR may serve as an early imaging marker for assessing the effectiveness of medical therapy.

**Figure 1:**
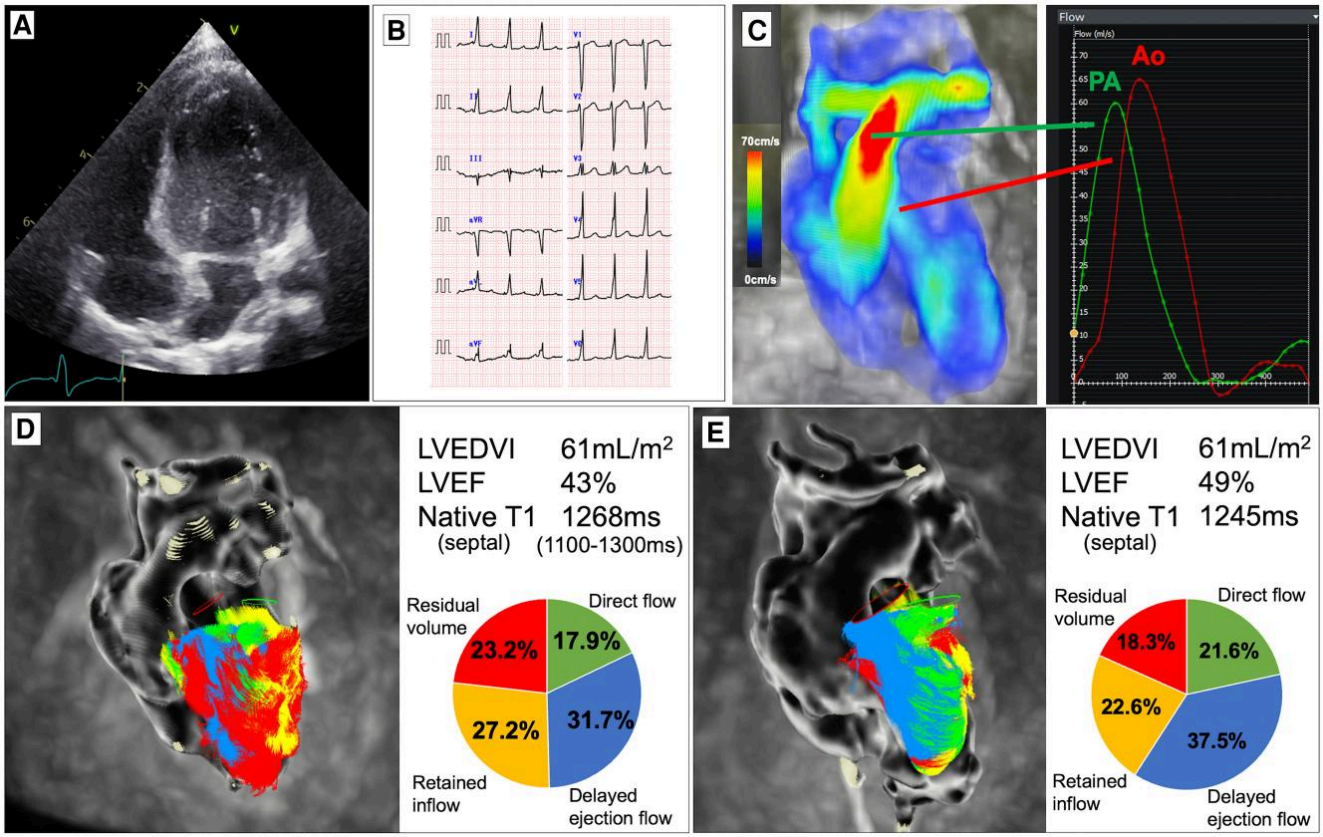
(*A*) echocardiography demonstrated paradoxical motion of the basal interventricular septum. (*B*) Electrocardiography demonstrated left bundle branch block, and a delta wave. (*C*) Pulmonary arterial ejection precedes aortic ejection on 4D flow CMR. (*D*, *E*) Intraventricular flow component ratios and LV function before and after treatment. Flow components were classified as direct flow (enters and exits the LV within one cardiac cycle), delayed ejection flow (already present in the LV that is ejected during systole), retained inflow (enters during diastole but remains after systole), and residual volume (remains within the LV for ≥2 cardiac cycles).^3^ Approximate adult reference values are: direct flow 37 ± 5%, delayed ejection flow 16 ± 3%, retained inflow 17 ± 4%, and residual volume 30 ± 5%.^3^ Flow component ratios improved after flecainide therapy. Ao, aorta; 4D flow CMR, Four-dimensional flow cardiac magnetic resonance; LV, left ventricular; LVEDVI, left ventricular end-diastolic volume index; LVEF, left ventricular ejection fraction; PA, pulmonary artery.

A 5-month-old boy was referred for evaluation of poor weight gain. Although his growth rate was within normal, echocardiography revealed left ventricular (LV) enlargement [end-diastolic diameter of 26.8 mm (108% of normal)] and reduced contraction (ejection fraction 42%). Paradoxical motion of the basal interventricular septum was also demonstrated (*Figure 1A*, Supplementary material online, *Videos S1*). Electrocardiography demonstrated sinus rhythm with left bundle branch block and delta waves, consistent with Wolff–Parkinson–White (WPW) syndrome type B (*Figure 1B*). No tachyarrhythmias were detected. Flecainide was used as first-line therapy for dyssynchrony related to accessory pathway conduction.

To further evaluate the effects of dyssynchrony on intracardiac flow patterns and ventricular mechanics, 4D flow cardiac magnetic resonance imaging (CMR) was performed. 4D flow CMR demonstrated a timing discrepancy between aortic and pulmonary ejection and intracardiac flow analysis showed a reduced direct-to-delayed ejection flow ratio, reflecting impaired ejection efficiency (*Figure 1C, D*, Supplementary material online, *Videos S2*). At 3-month follow-up, although no improvement was observed in the echocardiogram, electrocardiogram, and ejection timing, intracardiac flow component ratios improved (*Figure 1E*, Supplementary material online, *Videos S3*). With ongoing treatment, conventional findings also improved at 6-month follow-up (end-diastolic diameter of 28.1 mm, ejection fraction 57%).

To the best of our knowledge, this is among the first cases to use 4D flow CMR for ventricular dyssynchrony by measuring ejection time differences between the aorta and pulmonary artery. It also revealed changes in intracardiac flow patterns. Although catheter ablation is the standard treatment for LV dysfunction associated with WPW syndrome, flecainide is often used in children because catheter ablation may be technically challenging due to small body size.^1,2^ Flecainide may improve LV dyssynchrony by inhibiting accessory pathway conduction.^2^ These early effects may be reflected in changes in intracardiac flow patterns. Quantitative metrics derived from intracardiac flow components may provide valuable imaging biomarkers for monitoring therapeutic response in patients with WPW syndrome.

## Supplementary Material

ytag469_Supplementary_Data

## Data Availability

The data underlying this article will be shared on reasonable request to the corresponding author. This work was previously presented in part at the 29th Annual Scientific Sessions of the Society for Cardiovascular Magnetic Resonance (SCMR).

## Notes

**Statement of consent:** Written informed consent was obtained from the patient for publication of this case report in line with COPE guidance.
